# Sigmoid Volvulus as a Clinical Manifestation of Acquired Colonic Hypoganglionosis: A Case Report

**DOI:** 10.7759/cureus.33950

**Published:** 2023-01-18

**Authors:** Siraj Alsulimani, Noomen Haoues, Arwa M Aljuhani, Noor Fayoumi, Abdullah Al-Sawat

**Affiliations:** 1 Department of General Surgery, King Abdulaziz Specialist Hospital, Ta'if, SAU; 2 Department of Surgery, College of Medicine, Taif University, Ta'if, SAU

**Keywords:** sigmoid volvulus, pseudo-obstruction, constipation, ganglia, hirschsprung disease

## Abstract

A reduced and limited number of myenteric ganglia and low acetylcholinesterase activity in the lamina propria characterizes an unusual form of intestinal innervation disorder known as acquired or adult-onset hypoganglionosis. Only a few cases have been observed in adults, while the majority are diagnosed in infancy or youth. We report a rare case of colonic hypoganglionosis that presented as sigmoid volvulus in a 25-year-old female brought in to the ER. She underwent endoscopic decompression but developed a massive sigmoid volvulus with proximal colon dilatation. It was decided to do a total colectomy with an end ileostomy. The diagnosis was confirmed by histopathology, which revealed acquired hypoganglionosis. In order to prevent delayed or emergency presentation and the development of a stoma, the diagnosis of hypoganglionosis must be confirmed with full-thickness biopsies from all affected parts of the large bowel. Hypoganglionosis is rarely present, so young patients with a history of chronic constipation must be treated with a high index of suspicion.

## Introduction

Adult-onset or acquired hypoganglionosis (AHG) is an uncommon disorder affecting intestinal innervation. Its notable characteristics include small and few myenteric ganglia and poor acetylcholinesterase (AChE) activity in the lamina propria. Only a few occurrences have been documented in adults, and the majority of cases are diagnosed in infancy or childhood. All classified congenital intestinal innervation abnormalities have an incidence of 3-5% [1].

Intestinal innervation disorders include intestinal neuronal dysplasia, Hirschsprung disease (HD), hypoganglionosis, and ganglioneuromatosis [1]. Hypoganglionosis is common in females, with delayed symptoms, and carries a better prognosis compared to HD [2]. Differentiating between the types is difficult as they have the same clinical features: constipation or pseudo-obstruction [1,2]. For this reason, the differentiation is based on immunohistochemical examination and histological and electron microscopy findings through full-thickness biopsies for bowel segments [3].

Hypoganglionosis can be classified into: congenital and acquired, type I and type II, and isolated and associated with HD [2,4]. While the size of the ganglion cells in the congenital type does expand with age, the quantity does not, and both their size and their number are tiny at birth. The degeneration of ganglion cells, on the other hand, results in AHG [2]. Isolated hypoganglionosis (IHG), which composes 5% of all intestinal neuronal abnormalities, is rare [1,2,4]. In type I and type II hypoganglionosis, the distinction relates to the density and distribution of ganglion cells within the colonic plexuses, that is, severe ganglion reduction localized to a short segment or moderate reduction diffusing throughout the diseased colon [2]. Small and decreased number of myenteric ganglia, absence of or low AChE activity in the lamina propria, and hypertrophy of the muscularis mucosae, mainly in the colon and rectum, are the histopathological features of hypoganglionosis [4,5].

Only a few cases of the disease have been described in adulthood, and they are almost always discovered after elective surgery for pseudo-obstruction or chronic constipation [4]. In our report, the case of a 25-year-old female with hypoganglionosis and sigmoid volvulus is described.

## Case presentation

A 25-year-old female patient, with a history of one admission four months ago, was brought in through the ER due to sigmoid volvulus and presented with severe colicky abdominal pain associated with nausea and absolute constipation for one day. She had no other symptoms. She also had a history of chronic constipation as a normal bowel habit (bowel motion every three days).

**Figure 1:**
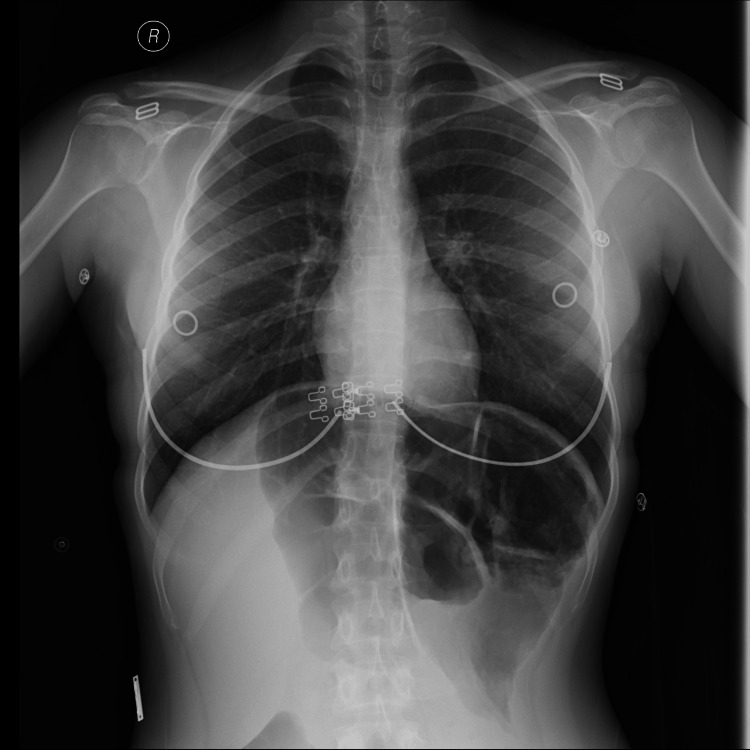
Upright chest x-ray suggesting dilated distal colon without pneumoperitoneum

**Figure 2:**
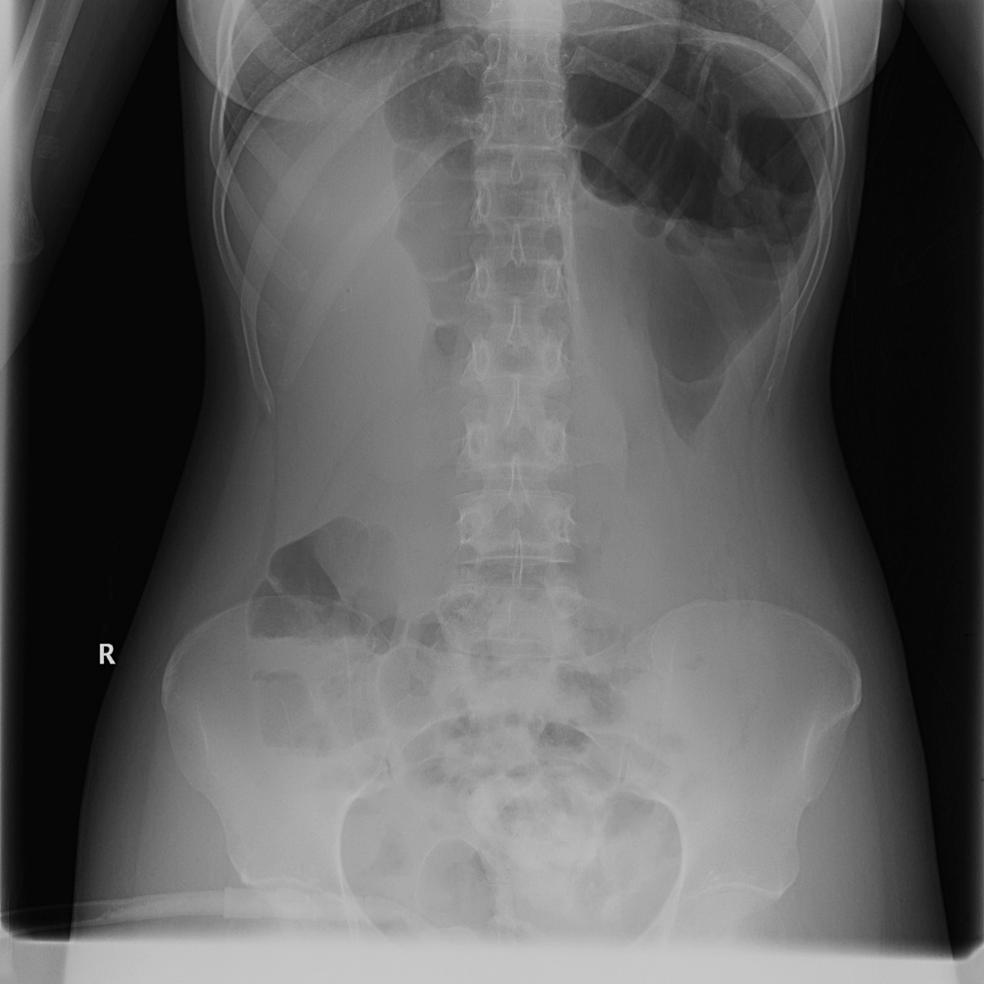
Plain x-ray abdomen suggesting dilated colon without small bowel dilation

On examination, she was conscious, oriented, vitally stable, and afebrile. Heart rate was 98 beats pr minute, blood pressure was 123/74, and temperature was 36.6 ^o^C. The abdomen was soft, lax, mildly tender on the left side, and hyper-resonant on percussion. Labs were within normal limits. X-ray showed dilated large bowel with a sign of sigmoid volvulus (Figures 1, 2). She underwent decompression sigmoidoscopy with rectal tube insertion, and her condition improved. She was advised and scheduled for elective laparoscopic sigmoidectomy after three days.

**Figure 3:**
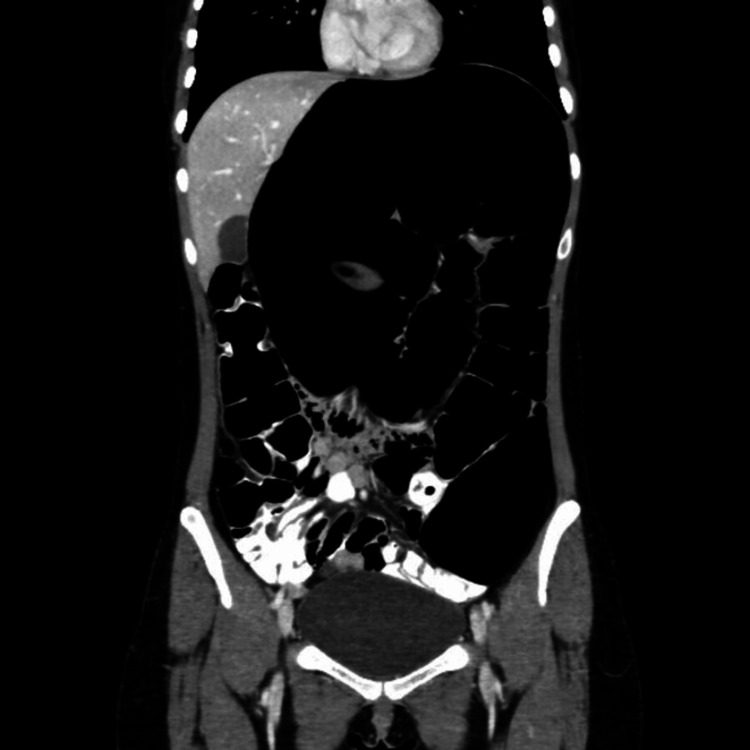
CT abdomen with IV and rectal contrast showing omega sign, suggesting sigmoid volvulus

On the third postoperative day after laparoscopic sigmoidectomy and colorectal anastomosis, when she started tolerating oral feeding, she developed severe abdominal pain, not relieved by analgesia. CT with IV and rectal contrast (Figure 3) showed dilated transverse colon with pneumoperitoneum. There was no evidence of leakage or collection. The patient underwent exploratory laparotomy with the following intraoperative findings: (i) dilated and non-healthy large bowel, (ii) leak from the anastomotic site, and (iii) pelvic localized collection. A total colectomy with end ileostomy was performed. The histopathology was consistent with acquired hypoganglionosis, confirming the diagnosis. The patient recovered well and was discharged on the ninth postoperative day.

## Discussion

The most common intestinal innervation disorder is HD, with a reported prevalence of 1: 5000 and a female-to-male ratio of 1: 4 [6]. Congenital lack of ganglion cells (aganglionosis) in the submucosal (Meissner's) and myenteric (Auerbach) neural plexuses of the gastrointestinal tract, starting from the rectal wall to variable lengths proximally, is what distinguishes it [7]. The cause of HD is the failure of craniocaudal migration of neuroblasts during the first three months of fetal development [7,8]. Despite the existence of ganglionic cells in the rectum, the presence of symptoms and signs that are comparable to the characteristics of HD, such as bowel obstruction, intestinal dilatation, and protracted constipation, characterize a disease category known as “allied diseases of HD”. It consists of seven illnesses, including (i) immaturity of ganglia, (ii) IHG, (iii) intestinal neuronal dysplasia (IND), (iv) megacystis micro-colon intestinal hypoperistalsis syndrome (MMIHS), (v) segmental dilatation of intestine, (vi) internal anal sphincter achalasia (IASA), and (vii) chronic idiopathic intestinal pseudo-obstruction (CIPO) [9].

As they have the same clinical features of bowel obstruction, intestinal dilatation, and chronic constipation, histological evaluation plays the most important role in the differentiation between them [10]. The other disorders are exceptionally rare, therefore when they are observed in a clinical setting, evidence should be taken into account and expert views should be sought [11]. Hypoganglionosis can be differentiated by the reduced number and size of myenteric ganglion cells in the “intramuscular layers” (Auerbach plexus), low or absent AChE activity in the lamina propria, and hypertrophy of the muscularis mucosa of the intestinal wall [3,9]. The number of myenteric ganglia should be less than one and a half per 10 mm or fewer than two neurons per ganglion of colon tissue [9,10].

In a review of the literature, it is seen that three findings are used to make the diagnosis: (i) There are just 40% as many nerve cells as in a normally innervated colon, (ii) the gap between ganglia is twice as wide, and (iii) the ganglions' average area is three times less than in a normally innervated colon [12-14].

IHG is a rare condition and accounts for 5% of all intestinal neuronal malformations [1,2,4]. It has been classified into congenital IHG (CIHG) and acquired IHG (AIHG). A survey performed in Japan identified 121 cases of CIHG and only nine cases of AIHG over 10 years [13]. In CIHG, the size and number of ganglions decrease at birth; as people mature, the size grows, but the number does not. The adult-onset hypoganglionosis or AIHG, on the other hand, is brought on by the degeneration of ganglion cells in nerve plexuses as a result of ischemia, intramural inflammation, viral infection, or autoimmune disease [2,14,15]. In type I and type II hypoganglionosis, the distinction relates to the density and distribution of the ganglion cells within the colonic plexuses, that is, severe ganglion reduction localized to a short segment or moderate reduction diffuse throughout the diseased colon [2]. Patients with type 1 (focal) hypoganglionosis show a transitional zone as focal narrowing with few ganglion cells, leading to a picture of functional obstruction. On the other hand, type 2 (diffuse) hypoganglionosis does not show a transitional zone with a diffuse reduction in ganglion numbers through the bowel.

The gold standard for definitive diagnosis of IHG is anorectal manometer, full-thickness bowel biopsies, and histological examination with histochemical staining [16,17], such as immunohistochemical C-kit, immunocytochemical (ICC), and silver staining. IHG is a disorder of neuromuscular junction, focusing on neural cell adhesion molecules and synaptophysin [18,19]. A study from South Korea reported 24 patients with hypoganglionosis who had undergone surgery for intractable constipation or chronic pseudoobstruction [12]. Few cases reported hypoganglionosis with an emergency presentation [4,20]. Our patient had megacolon and sigmoid volvulus impending perforation; therefore, she underwent elective total colectomy with end ileostomy. Waiting for stoma closure for either ileorectal or ileoanal anastomoses depends on full-thickness rectal biopsies.

## Conclusions

Young individuals with a history of chronic constipation should be addressed with a high index of suspicion since hypoganglionosis is exceptionally rare. It should be more investigated and full-thickness biopsies taken from all affected parts of the large bowel to confirm the diagnosis to avoid delayed or emergency presentation and avoid stoma creation. Histopathology allows differentiation among the variants of hypoganglionosis (innervation disorders). The awareness of this rare disease can help in enhancing clinical judgment, treating patients, and collecting evidence.
